# The Texas Homœopathic Pellet

**Published:** 1883-12

**Authors:** 


THE TEXAS HOMŒOPATHIC PELLET was started in August last by Dr. C. E. Fisher, assisted by Drs. A. P. Davis and J. G. Bowen. *The Pellet* is to be devoted to "the missionary interests of Homœopathy," whatever *they* may be. We know of no better place for missionary effort than Texas, medically and morally, and so we wish *The Pellet* God-speed in its work.

